# Prevalence of Fabry Disease among Patients with Parkinson's Disease

**DOI:** 10.1155/2022/1014950

**Published:** 2022-01-24

**Authors:** Alexandra Lackova, Christian Beetz, Sebastian Oppermann, Peter Bauer, Petra Pavelekova, Tatiana Lorincova, Miriam Ostrozovicova, Kristina Kulcsarova, Jana Cobejova, Martin Cobej, Petra Levicka, Simona Liesenerova, Daniela Sendekova, Viktoria Sukovska, Zuzana Gdovinova, Vladimir Han, Mie Rizig, Henry Houlden, Matej Skorvanek

**Affiliations:** ^1^Department of Neurology, University of Pavol Jozef Šafárik, Košice, Slovakia; ^2^Department of Neurology, University Hospital of L. Pasteur, Košice, Slovakia; ^3^CENTOGENE GmbH, Rostock, Germany; ^4^University College London, Institute of Neurology, Department of Neuromuscular Disorders, Queen Square, WC1N 3BG London, UK

## Abstract

**Background:**

An increased prevalence of Parkinson's disease (PD) disease has been previously reported in subjects with Fabry disease (FD) carrying alpha-galactosidase (GLA) mutations and their first-line relatives. Moreover, decreased alpha-galactosidase A (AGLA) enzymatic activity has been reported among cases with PD compared to controls.

**Objective:**

The aim of our study was to determine the prevalence of FD among patients with PD.

**Methods:**

We recruited 236 consecutive patients with PD from February 2018 to December 2020. Clinical and sociodemographic data, including the MDS-UPDRS-III scores and HY stage (the Hoehn and Yahr scale), were collected, and in-depth phenotyping was performed in subjects with identified GLA variants. A multistep approach, including standard determination of AGLA activity and LysoGb3 in males, and next-generation based GLA sequencing in all females and males with abnormal AGLA levels was performed in a routine diagnostic setting.

**Results:**

The mean age of our patients was 68.9 ± 8.9 years, 130 were men (55.1%), and the mean disease duration was 7.77 ± 5.35 years. Among 130 men, AGLA levels were low in 20 patients (15%), and subsequent Lyso-Gb3 testing showed values within the reference range for all tested subjects. In 126 subsequently genetically tested patients, four heterozygous p.(Asp313Tyr) GLA variants (3.2%, MAF 0.016) were identified; all were females. None of the 4 GLA variant carriers identified had any clinical manifestation suggestive of FD.

**Conclusions:**

The results of this study suggest a possible relationship between FD and PD in a small proportion of cases. Nevertheless, the GLA variant found in our cohort is classified as a variant of unknown significance. Therefore, its pathogenic causative role in the context of PD needs further elucidation, and these findings should be interpreted with caution.

## 1. Introduction

Fabry disease (FD) belongs to the group of genetically determined forms of lysosome storage disorders with X-linked inheritance, which lead to a deficiency of lysosomal enzyme–alpha-galactosidase A (AGLA), resulting in the accumulation of glycosphingolipids, especially globotriaosylceramide (Gb3) in vital organs [1]. Although FD is a disease with X-linked inheritance, clinical symptoms are very common in females as well. Some mutations cause classic disease, while others result in a milder disease phenotype with later onset [2]. The clinical spectrum of the disease is broad. Early signs include typical neurological manifestations, skin changes, renal involvement, and characteristic cardiovascular manifestations. Due to the complexity of the disease, multidisciplinary management is needed [3]. As for the group of lysosomal storage diseases, the association between mutations in glucocerebrosidase (GBA), which encodes the lysosomal enzyme glucocerebrosidase (GCase), and Parkinson's disease (PD) has highlighted the importance of lysosomal function in PD pathogenesis [4]. Since lysosomes are involved in the process of alpha-synuclein degradation, it is thought that their dysfunction may lead to its accumulation and subsequent PD formation [5]. In recent years, there have been expanding studies on the interrelationship between parkinsonism and the mutation of the alpha-galactosidase gene (GLA) gene in FD. An increased prevalence of PD disease has been previously reported in subjects with FD and their first-line relatives [6]. Moreover, decreased AGLA enzymatic activity has been reported among cases with PD compared to controls [2]. This points to a potential relationship between these two disorders. The function of the AGLA enzyme represents a new therapeutic approach for genetically determined PD, as increasing the levels of these enzymes leads to a reduction in the levels of alpha-synuclein [7]. Nevertheless, the prevalence of FD among PD patients has not been systematically studied so far. Therefore, we aimed to determine the prevalence of FD among patients with PD in a single tertiary movement disorders centre in Kosice, Slovakia.

## 2. Methods and Participants

### 2.1. Participants and Clinical Evaluation

Overall, from February 2018 to December 2020, we recruited 236 consecutive patients with PD diagnosed based on the MDS clinical criteria for PD [8] in a single tertiary movement disorders center in Kosice, Slovakia, irrespective of their ethnicity, disease duration, disease stage, age of onset, or cognitive status. Several aspects were assessed: a—motor examination was performed using the Movement Disorder Society-Unified Parkinson's Disease Rating Scale (MDS-UPDRS) [9], including the Hoehn and Yahr (HY) scale to assess the disease stage. The MDS-UPDRS is a four-subscale combined scale that comprehensively assesses the symptoms of PD. It consists of the following: Part I—nonmotor experiences of daily living, Part II—motor experiences of daily living, Part III—motor examination, and Part IV—motor complications. All items are scored on a scale from 0 (normal) to 4 (severe), and the total score for each part is obtained from the sum of the corresponding item scores.

Basic sociodemographic data were recorded, as well as the age of onset and disease duration, disease subtype (tremor-dominant, akinetic-rigid, mixed, and PIGD [10]), and information about family history of neurodegenerative parkinsonism. The presence of selected clinical parameters was evaluated, including dementia, based on the Montreal Cognitive Assessment (MoCA) [11] and the Parkinson's Disease-Cognitive Rating Scale (PD-CRS) [12], both recommended for cognitive screening in PD by the Movement Disorder Society [13]. MoCA is a one-page cognitive impairment test assessing multiple cognitive domains such as short-term memory, executive functions, visuospatial abilities, naming, attention, and working memory, language, concentration, verbal abstraction, and orientation, with a maximum score of 30 points and cutoffs of 25/26 points for PD-MCI (PD-mild cognitive deficit) (sensitivity: 90%; specificity: 75%) and 20/21 points for PD-D (PD-dementia) (sensitivity: 81%; specificity: 95%) [14]. PD-CRS is a new battery of cognitive scales to assess cognitive decline in PD patients. This battery consists of subtests to assess cortical (naming confrontation and copying clocks) and subcortical functions (sustained attention, working memory, alternating and action verbal fluency, drawing clocks, immediate and delayed verbal memory with free recall) using a total of 9 tasks. The maximum subcortical and cortical function scores are 104 and 30, with a cutoff score of ≤81 points showing a sensitivity of 79% and specificity of 80% for PD-MCI compared with healthy controls [15], and cutoffs of both ≤62 and ≤64 points with a sensitivity and specificity of ≥94% for PD-D compared with healthy controls [16].

The study was performed according to the Declaration of Helsinki (1975); it was approved by the local ethics committee and all patients signed the written informed consent before enrolment.

### 2.2. Enzymatic Activity Assay

The following method was applied for the determination of AGLA (alpha-galactosidase) activity in DBS (dried blood spots). The used protocol implies extraction of the AGLA from DBS, incubation with a synthetic substrate for a defined amount of time, and detection of the enzymatic product using fluorimetry. AGLA enzymes are extracted from dry blood spots in sodium acetate buffer in a 96-well plate at 37°C with agitation. On top of the extracts, a specific synthetic substrate (4-methylumbelliferyl-*α*-D-galactopyranoside) is added, and the plates are incubated at 37°C for 4–6 h. The reaction is stopped by adding carbonate buffer (changing the pH to 10.7). The quantitation of the product (4-MU) was performed by fluorimetry on a Victor2 Fluorometer (PerkinElmer) using an external calibration line of 4-methylumbelliferone. The results of the enzymatic activity determination were calculated in *μ*mol/L/h. Enzymatic AGLA levels were not analyzed in females as previous studies have shown an inconsistent relationship between normal AGLA levels and GLA mutation status in FD subjects. On the other hand, normal AGLA levels in men predict a normal GLA mutation status, and thus, further Lyso-Gb3 level determination and genetic studies were performed in men only in the case of abnormal AGLA levels.

### 2.3. Determination of the Levels of Lyso-Gb3

Lyso-Gb3 levels were determined only in men with abnormal AGLA levels. For each sample, three 3.2 mm punches from a dried blood spot are treated with 150 *μ*L metabolite extraction buffer (50 *μ*L DMSO/water 1/1 and 100 *μ*L IS solution in ethanol) at 37°C for 30 min. Potential filter card-derived debris is removed in a subsequent filtration step. The metabolite of interest is eventually quantified by liquid chromatography multiple-reaction-monitoring mass spectrometry (LC/MRM-MS) coupled with ultraperformance liquid chromatography (UPLC). Absolute concentrations are calculated based on an intraexperimental calibration line.

### 2.4. Genetic Studies

**Figure 1:**
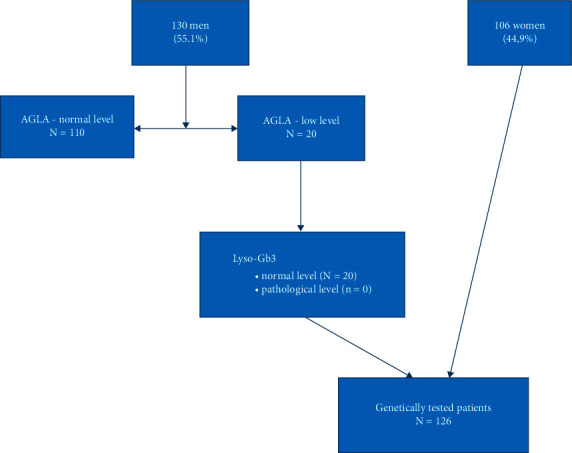
Algorithm of laboratory testing in male and female subjects with PD.

Genetic studies were performed in all females and in males with abnormal AGLA levels, as shown in the flowchart in Figure 1. The coding sequence of GLA along with at least 50 base-pairs of neighbouring intronic or UTR sequence was analyzed by a diagnostically validated in house assay as described in more detail previously [17].

### 2.5. Statistical Analysis

SPSS Inc. statistical software version 22.0 (Chicago, IL, USA) was used for statistical analysis. First, we described the basic sociodemographic characteristics of our study group. Subsequently, laboratory parameters and the prevalence of mutations in the GLA gene were analyzed. Lastly, we described the clinical characteristics of subjects with identified GLA variants.

## 3. Results

The study included 236 PD patients with a mean age of 68.9 ± 8.9 years, of which 130 (55.1%) were male and the mean disease duration was 7.77 ± 5.35 years. Detailed characteristics of the PD sample are described in Table 1. Among 130 men, AGLA levels (with an average value of 22.4 ± 7.57 *μ*mol/L/h) were low in 20 patients (15%) (with an average value of 12.25 ± 2.51 *μ*mol/L/h) and subsequent Lyso-Gb3 testing showed values within the reference range for all tested subjects (0.9–1.7 ng/mL). In 126 genetically tested patients (20 males with low AGLA levels and 106 females), four c.937G > T, p. (Asp313Tyr) variants were identified. Interestingly, all of the positive patients were women, and none of them reported a family history of PD. The age of onset for all subjects carrying heterozygous GLA p.Asp313Tyr variants was >55 years, and most had a mixed PD phenotype (3/4). All GLA p.Asp313Tyr positive subjects had a good response to dopaminergic medication, and all of them developed fluctuations and dyskinesia at the time of clinical assessment. In terms of nonmotor symptoms, all patients complained of autonomic dysfunction, especially urinary problems (3/4 had urine urgency), and the majority of them also complained of sleep-related issues, fatigue (3/4), and mood disorders (3/4) such as depression and anxiety. Cognitive status was normal in 2 GLA p.Asp313Tyr positive subjects according to MoCA and PD-CRS, while 2 patients scored in the PD mild cognitive impairment range. In terms of prodromal features, all four patients carrying the p.(Asp313Tyr) complained of increased sweating and three patients complained of smell loss; however, none of them reported symptoms of REM-sleep behavior disorder (RBD), and only one reported constipation.

All patients, except one who refused, underwent brain MRI examinations, which revealed only mild subcortical white matter T2 hyperintensities, likely of vascular etiology. In one of the subjects, a swallow-tail sign at the level of substantia nigra was revealed. A DaT scan was performed on all 4 subjects with pathological findings.

All patients carrying the p.(Asp313Tyr) variant underwent cardiological (including echocardiography), nephrological, and ophthalmological examinations, which were without any pathological findings that would support the diagnosis of FD. Detailed clinical characteristics of patients harboring p.(Asp313Tyr) mutations in our cohort are summarized in Table 2.

## 4. Discussion

Genetic screening of 127 PD patients for GLA variants previously associated with FD in our cohort resulted in the identification of 4 heterozygous GLA p.(Asp313Tyr) variants. The GLA p.Asp313Tyr allele frequency of 1.6% found in our PD population was significantly higher compared to the allele frequency in the general population reported in the major genetic databases ExAC (0.3%), gnomAD (0.3%), TOPMed (0.3%), or 1000 Genomes Project (0.2%) [18]. To the best of our knowledge, this is the first study surveying the prevalence of FD among patients with PD.

The exact etiology of PD is still not known. Several molecular pathways have been associated with the pathology of neurodegeneration, including mitochondrial dysfunction [19], oxidative and proteolytic stress, immune and inflammation responses [20], and the autophagy-lysosomal pathway (ALP) [21]. The role of lysosomal dysfunction in the pathogenesis of parkinsonism is highlighted by the link between PD and heterozygous GBA carriers [6]. Although there is considerable literature supporting a relationship between GBA and synucleinopathies [4, 22, 23], not much is known about a possible association between synucleinopathies and FD, which may also be common [24, 25]. Parkinsonism has been observed in patients with FD in several studies [6, 26–29] which suggests that there may be an increased risk of developing PD in individuals carrying GLA mutations. Recent findings of decreased AGLA, which are characteristic of FD in some patients with parkinsonism, suggest a link between the two diseases [2]. Due to these findings, we surveyed patients with PD to determine the prevalence of FD. In four patients with PD who were genetically tested for FD, the p. (Asp313Tyr) variant was identified, the allele frequency of which was higher than in the general population (1.6% vs. 0.2–0.3%). Interestingly, all patients were women and were carrying the same p. (Asp313Tyr) GLA variant. Previous studies reporting on the clinical features of parkinsonism in the few described FD subjects [27–30] showed a typical age of onset, mostly an akinetic-rigid type of parkinsonism and typically a good response to dopaminergic treatment. Prodromal and nonmotor symptoms were less described in these reports. This is in line with our findings showing a typical later onset of PD, with a good therapeutic response to therapy, where nonmotor symptoms were dominated by autonomic symptoms as described previously. None of the previous reports or our own data, thus far, supports the presence of clinical features of parkinsonism that would be suggestive of GLA carrier status.

According to the existing literature, more than 900 variants in the GLA gene have been described [31], but the specific clinical impact of most mutations has not yet been well explored [32]. Although substantial literature exists, it is still under debate whether the p. (Asp313Tyr) variant represents a disease-causing mutation, a low-pathogenic variant, or just a polymorphism [33]. In 1993, this mutation was reported as causative in a male patient with a classical manifestation of FD [34]. However, 10 years later, a more detailed analysis revealed a second missense mutation, p. (Cys172Gly), in the same patient, which questioned the pathogenicity of the p. (Asp313Tyr) variant [35]. Later, the mutation was identified as a “pseudodeficient allele,” suggesting that mutant enzyme activity is pH dependent [35, 36]. Similarly, Niemann et al. and Oder et al. describe this variant as not clinically relevant [37] or as nonpathogenic for FD [32], and the p. (Asp313Tyr) variant has also been referred to as a polymorphism [38]. The possible pathological significance of the p.(Asp313Tyr) variant in the GLA gene has not been verified in the Hasholt study during the examination of members of two Danish families. His findings only support the assumption that p.(Asp313Tyr) is a rare variant without pathological significance [39].

These findings contradict other studies showing that the p. (Asp313Tyr) variant may lead to FD-nervous system manifestations [33, 39–41]. Koulousios et al. have shown the possibility that the p. (Asp313Tyr) variant might be considered as disease causing by finding that the prevalence of the p. (Asp313Tyr) variant among patients with FD is more than 35%, while the frequency in the general population is estimated to be less than 1% [41]. According to Koulousios et al., this variant is associated with a milder phenotype and a later onset of the disease [41]. Data in the Moulin study showed that the p. (Asp313Tyr) GLA variant may lead to symptoms and organ manifestations compatible with FD [40]. Also, Zompola et al. reported two newly diagnosed Fabry cases with nervous system manifestations related to the p.(Asp313Tyr) variant, which strengthened the presumption of the pathogenicity of this mutation [33]. In a recent review of Effraimidis et al., the authors stated that the frequency of the p. (Asp313Tyr) variant in comparison to the general population is only higher in neurologic disorders [42]. In fact, patients carrying GLA variants may be asymptomatic or show a spectrum of mild clinical manifestations, including cerebrovascular disease, such as the recently reported cerebral hemodynamic changes in asymptomatic FD subjects at risk for cerebrovascular events [43]. Preclinical detection of neurovascular involvement in FD might allow appropriate management and prevention of future cerebrovascular complications and disability [43]. Due to the findings stated previously, patients carrying the p.(Asp313Tyr) variant should be monitored annually, as enzyme replacement therapy could be used if organ manifestations occur [33].

In summary, this study showed a higher incidence of the p. (Asp313Tyr) variant with a higher prevalence than in the general population, further emphasizing the importance of the autophagy-lysosomal pathway study in PD. None of our four patients showed cardiac hypertrophy, renal dysfunction, acroparesthesias, or corneal opacities. MRI did not reveal morphological or functional abnormalities specific for FD. Overall, the results of this study suggest that the p. (Asp313Tyr) variant leads to a nonpathological or very mild variant of FD at most. On the other hand, this mutation may not always be reflected in the fully expressed FD phenotype and may not be accompanied by low levels of AGLA, as the residual activity of this enzyme is preserved [41]. Also, in the context of PD and FD, this variant of unknown significance may help clinicians and researchers in questioning the causative role of genetic variants within the daily clinical and diagnostic settings [44]. Accordingly, as stated, its pathogenic, causative role in the context of PD needs to be further elucidated, and these findings should be interpreted with caution. Observing the four female patients as the asymptomatic carriers of p. (Asp313Tyr), and especially their sons, is needed.

In addition, the range of the Fabry phenotype in women can vary from asymptomatic to severely affected compared to men, where the phenotype is usually more severe [2]. The diagnosis of this disease is challenging because the phenotypic manifestation of FD can range from typical manifestations of the disease to unusual and incorrect, and late diagnosis can delay the necessary treatment [32]. Specific treatment for orally administered *α*-galactosidase A inhibitor or recombinant human alpha-galactosidase (ERT) is available, with better results if therapy is initiated before organ damage [2]. With the perspective of potential FD therapy as a disease modifier also for PD, screening for prodromal symptoms of PD is appropriate.

### 4.1. Strengths and Limitations

One of the limitations of this study is the lack of an ethnically-matched healthy control group, which does not allow a direct comparison of prevalence between Slovak PD subjects and healthy controls, although the allele frequency of the GLA p.Asp313Tyr variant in major genetic databases is rather constant at the level of 0.2–0.3%. Also, as part of the clinical lab protocol, enzymatic AGLA levels were not analyzed in females as previous studies have shown an inconsistent relationship between normal AGLA levels and GLA mutation status in FD subjects. On the other hand, normal AGLA levels in men predict a normal GLA mutation status, and thus, genetic analyses were performed in men only in the case of abnormal AGLA levels. One other limitation is that our cohort includes a wider range of age of onset of disease of our patients, taking into account that most of the young-onset PD are genetic, alternative etiologies of PD besides FD can explain the final prevalence.

## 5. Conclusion

While the prevalence of the GLA p.Asp313Tyr variant seems to be higher in PD patients compared to the general population, its pathogenic causative role in the context of PD needs to be further elucidated, and these findings should be interpreted with caution. The clinical significance of the variant abovementioned is still under debate. Patients carrying the p.(Asp313Tyr) variant should be monitored annually, as enzyme replacement therapy could be used if organ manifestations occur.

## Figures and Tables

**Table 1:** Characteristics of the PD population (*N* = 236).

	Mean ± SD (*N*, %)	Range
Age (years)	68.9 ± 8.9	33–88
Gender		
Male	130 (55.1%)	
Female	106 (44.9%)	
Age of onset	61.13 ± 10.35	27–85
Disease duration	7.77 ± 5.35	0–30
MDS-UPDRS part III score	27.58 ± 13.35	3–80
Presence of GLA gene mutations (*N* = 126) c.937G > T, p. (Asp313Tyr)	4 (3.17%)	
alpha-Galactosidase level in men (*N* = 129) <15.3 *μ*mol/L/h (pathological) Pathological level of AGLA	22.40 ± 7.57 20 (15%) 12,25 ± 2.51	5.3–57.2 5.3–15.2
Lyso-Gb3 level in men > 1.8 ng/ml (pathological)	1,23 ± 0,22 0	0.9–1.7

MDS-UPDRS: Movement Disorder Society-Unified Parkinson's Disease Rating Scale.

**Table 2:** Detailed clinical characteristics of PD patients harbouring the p.(Asp313Tyr) variant.

GLA variant	Origin	Family history	Age	Gender	Age of onset	PD subtype	Falls	Freezing	Tremor	MDS-UPDRS part III ON	HY stage	Motor fluctuations	NMS	Cognition	Initial motor features	Prodromal features	Current medication
p.(Asp313Tyr)	Slovak (ES)	−	68	F	56	PIGD	+	+	−	35	4	Wearing off, PoD dyskin	Insomnia, fatigue, daytime sleepiness, frequent Urination, nocturia, constipation, depression, apathy, anxious mood, vertigo-dizziness,	MoCA 29/30, PD-CRS 99/134	Balance problems, left-side bradykinesia, and rigidity	Smell loss, sweating	LCIG 4,2 ml/per hour
p.(Asp313Tyr)	Slovak (VA)	−	66	F	61	Mixed	−	−	+	13	1	PoD dyskinesia, wearing off	Urine urgency, nykturia, fatigue, mild anxious mood, mild hyposmia	MoCA 27/30, PD-CRS 94/134	Pain and tremor of the left hand	Fatigue, constipation, sweating, urine urgency, anxiety	L/C 200 mg/D
p.(Asp313Tyr)	Slovak (AS)	−	75	F	69	Mixed	−	+	−	20	2	PoD dyskinesia	Daytime sleepiness, fatigue, urine urgency	MoCA 25/30, PD-CRS 62/134	Tremor of the right hand	Smell loss, excessive sweating	L/C 875 mg/D PPX 1,57 mg/D
p.(Asp313Tyr)	Slovak (HR)	−	70	F	60	Mixed	−	+	+	32	2	PoD dyskinesia, wearing off	Urine urgency, depression, anxious mood, chronic pain of lower limbs	MoCA 21/30	Tremor of the right hand	Sweating, urine urgency	L/C 500 mg/D rasagilin 1 mg/D

Y, years; PD, Parkinson's disease; MDS-UPDRS, Movement Disorder Society-Unified Parkinson's Disease Rating Scale; HY, Hoehn and Yahr; NMS, nonmotor symptoms; F, female; M, male; PIGD, postural instability gait disorder; PoD, peak-of-dose; MoCA, Montreal Cognitive Assessment; L/C, levodopa/carbidopa; PPX, pramipexole; PD-CRS, and Parkinson's disease cognitive rating scale.

## Data Availability

Data are available from the corresponding author upon individual request.
